# First person – Tamara González-Costa

**DOI:** 10.1242/dmm.053076

**Published:** 2026-07-22

## Abstract

First Person is a series of interviews with the first authors of a selection of papers published in Disease Models & Mechanisms, helping researchers promote themselves alongside their papers. Tamara González-Costa is first author on ‘ Systemic and cardiac pathology induced by a clinically relevant *USP8* activating mutation’, published in DMM. Tamara is a postdoctoral researcher in the lab of José Luis de la Pompa at Centro Nacional de Investigaciones Cardiovasculares Carlos III, Madrid, Spain, investigating the cellular and molecular mechanisms that drive cardiovascular development and disease.

**Figure:**
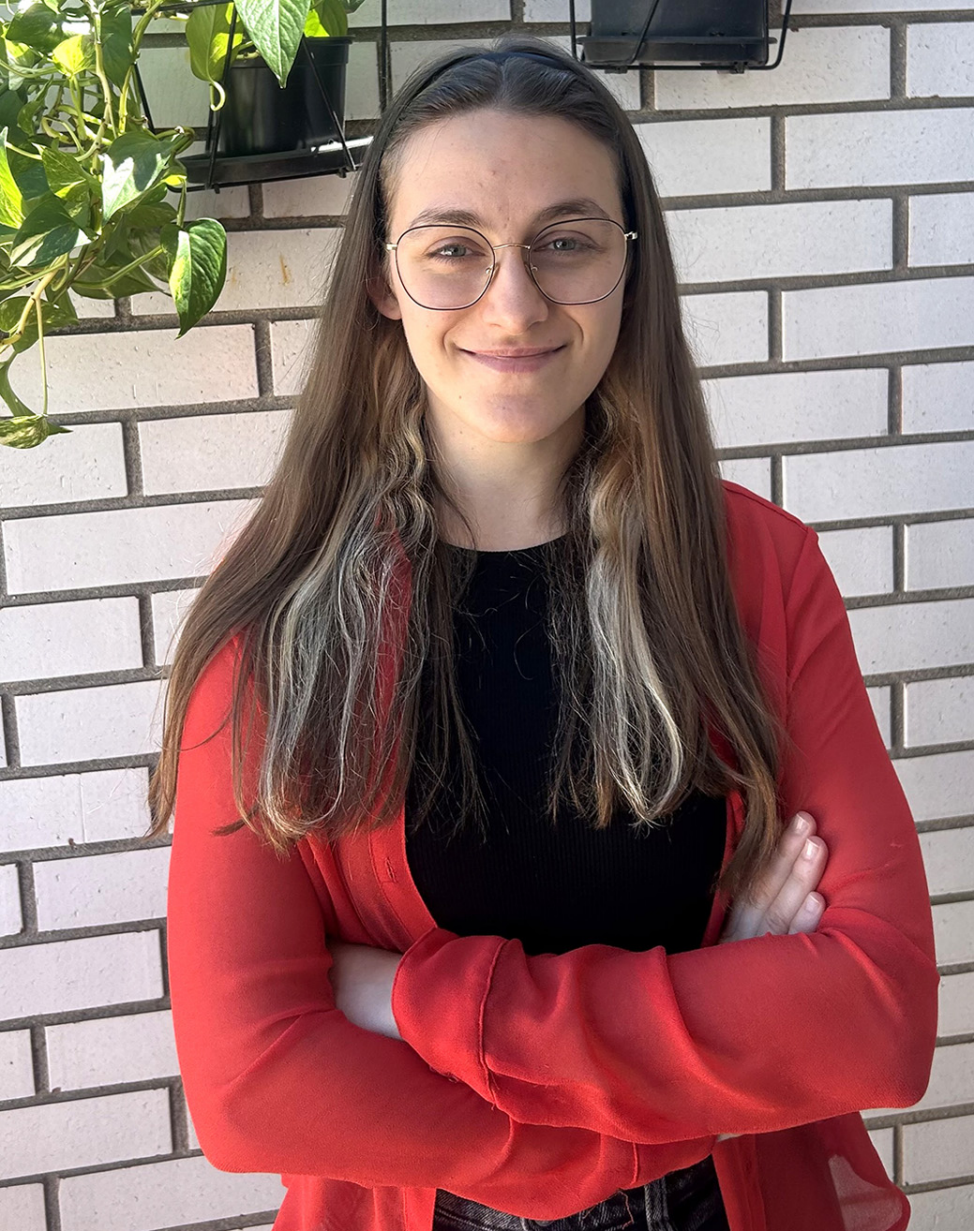
Tamara González-Costa

**Who or what inspired you to become a scientist?**

I have always been curious and fascinated by learning new things. In high school, I was especially drawn to biology, chemistry and philosophy, as they tried to answer the kind of questions I found myself asking. During my BSc, I joined a lab in the summer and began investigating developmental genetics, which sparked a strong interest in developmental biology. I was fascinated by how a complex living organism arises from a single cell, and how development proceeds with such remarkable precision. At the same time, I became aware that many developmental processes can go wrong, leading to severe congenital malformations. This motivated me to pursue a PhD with the aim of channelling my curiosity into something purposeful, with the potential to improve healthcare. And this is how I ended up investigating congenital heart disease and cardiovascular disease.

Our results suggest that mutated USP8 is not just a driver of pituitary tumours and hypercortisolism, but a direct cause of systemic pathology in a USP8-associated Cushing's syndrome

**What is the main question or challenge in disease biology you are addressing in this paper? How did you go about investigating your question or challenge?**

Cushing's disease is a rare endocrine disease that originates from a corticotroph pituitary adenoma that drives excessive cortisol production and leads to Cushing's syndrome. The syndrome includes a range of symptoms including heart, bone, muscle and eye problems, which persist even after cortisol levels are normalized. Somatic mutations in ubiquitin-specific protease 8 (*USP8*) are found in pituitary adenomas of human patients, and a *de novo* germline mutation was identified in a patient who exhibited not only Cushing's disease but also atypical systemic symptoms. Our main question was whether mutated USP8 has direct effects on the pathophysiology of the syndrome, beyond pituitary involvement and independently of cortisol. To address this, we generated a conditional transgenic mouse model expressing the most common human USP8 mutation. We tested systemic expression and, independently, myocardium-specific expression, which allowed us to dissect tissue-specific pathogenic mechanisms of USP8. We show that these transgenic mice develop cardiac, musculoskeletal and eye disease, even with normal cortisol levels.

**How would you explain the main findings of your paper to non-scientific family and friends?**

In some patients with Cushing's disease, a protein called USP8 is mutated in the pituitary gland, which leads to excessive cortisol hormone release and, as a consequence, health problems in other organs such as the heart, bones, muscles and eyes. We wanted to know if this mutated protein can damage these other organs directly, not just by changing hormone levels at the pituitary. For that, we generated mice that carry the most common human USP8 mutation. These mice developed muscle weakness, bone problems, eye disease with cataracts and heart disease, even though their hormone levels were mostly normal. When we located the mutation only in the heart muscle of mice, the same heart problems appeared. Our study shows that the mutated USP8 protein can directly harm organs like the heart, skeletal muscle, bones and eyes, beyond its effect on the pituitary and hormones.

**What are the potential implications of these results for disease biology and the possible impact on patients?**

Our results suggest that mutated USP8 is not just a driver of pituitary tumours and hypercortisolism, but a direct cause of systemic pathology in a USP8-associated Cushing's syndrome. Importantly, our mouse model does not fully recapitulate the classic endocrine features of Cushing's disease, such as corticotroph adenomas or sustained hypercortisolism, yet it recapitulates key cardiac, musculoskeletal and ocular features. This supports the idea that the clinically relevant *USP8* activating mutation has conserved roles in maintaining homeostasis of the heart, skeletal muscle, bones and eyes, independently of cortisol. In addition, our model provides a genetic tool to further investigate the pathophysiology of this disease and the stratification of patients, and to design specific therapies.

**Figure:**
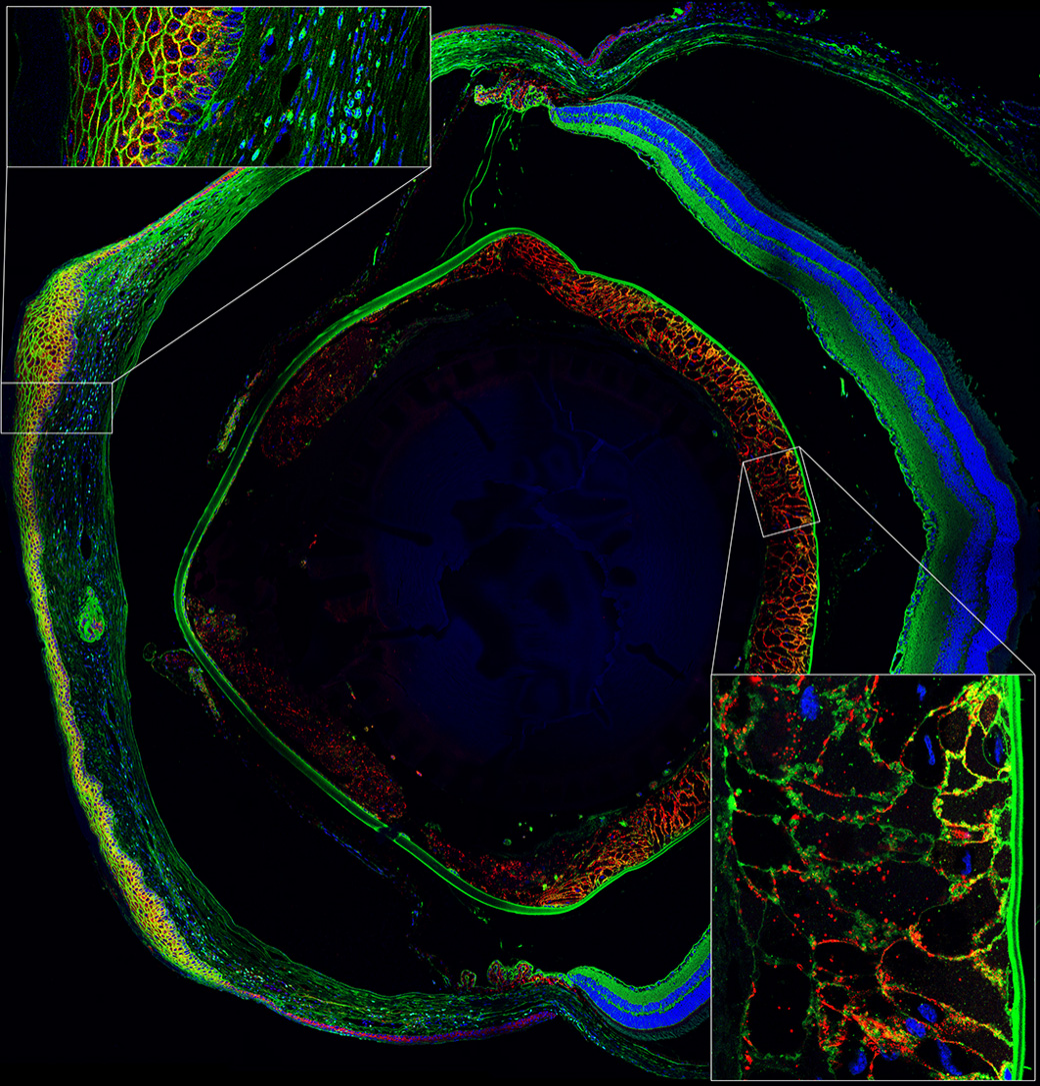
**Diseased eye showing corneal defects (upper inset) and cataracts (lower inset).** Transgenic mice expressing a clinically relevant USP8 mutation develop epithelial corneal thickening and hypertrophy, with membranes labelled with wheat germ agglutinin (green), along with vascularization, indicative of a chronic wound-healing response. In the lens, severe cataracts are associated with disorganized fibre membranes and with mislocalization and accumulation of the gap junction protein connexin 43 (red). Nuclei are stained with DAPI (blue).

**Why did you choose DMM for your paper?**

DMM is a well-known and respected Open Access journal that provides an excellent platform for studies that investigate the biological mechanisms of disease, so it was a perfect fit for our work. I also value The Company of Biologists' dedication to supporting and inspiring the biological community.

… trying to maintain high scientific productivity and learn to secure funding while keeping a work–life balance can lead to burnout

**Given your current role, what challenges do you face and what changes could improve the professional lives of other scientists in this role?**

As a postdoctoral researcher, I think the main challenge is instability and uncertainty. You need to produce high-quality research while also securing funding for your own position and projects. This is stressful and distracting. You learn to multitask, but trying to maintain high scientific productivity and learn to secure funding while keeping a work–life balance can lead to burnout. I believe it is important to establish better conditions to retain talent in academia. The system urgently needs to offer more stability to early-career researchers.

**What's next for you?**

At the moment, I am looking for a postdoctoral position abroad in cardiac biology and regeneration, while I am completing a specialization diploma in bioinformatics. In my next position, I am eager to integrate experimental and computational approaches to address biological questions. I believe biomedical research is already evolving in this direction, bridging wet-lab and data-driven research, and I do believe this integration is very powerful.

**Tell us something interesting about yourself that wouldn't be on your CV**

Outside of the lab, I stay active through sports. It was never my thing as a child, but I am embracing it in my adulthood. It helps me to stay healthy both physically and mentally. I always enjoy a good run after work; it really helps me to clear and unfog my mind. I am also learning aerial silks, which is very challenging but also fun! I also enjoy learning new languages. I am currently studying Japanese and German, although I do mention this on my CV.
